# Comparative Analysis of the Modified Aldrete Score and Fast-Track Criteria for Post-general Anaesthesia Recovery: A Narrative Review

**DOI:** 10.7759/cureus.64439

**Published:** 2024-07-12

**Authors:** Janhavi S Dahake, Neeta Verma

**Affiliations:** 1 Anesthesiology, Jawaharlal Nehru Medical College, Datta Meghe Institute of Higher Education and Research, Wardha, IND

**Keywords:** post-operative nausea and vomiting (ponv), post-operative recovery, fast track criteria, modified aldrete score, anaesthesia

## Abstract

There are two commonly used scoring systems to evaluate recovery from general anaesthesia (GA): the Modified Aldrete Score (MAS) and the Fast-Track Criteria (FTC). Recently, concerns have been expressed about the safety and effectiveness of the Aldrete scoring system due to its exclusion of an assessment for pain or nausea, which can exacerbate recovery from surgery and anaesthesia and cause many patients to experience these side effects. FTC was created to evaluate post-operative nausea vomiting, and pain in order to assess recovery from GA. More data are needed to compare these scoring criteria in low-income countries like India. Understanding how these scores can be effectively utilised in our settings is crucial for ensuring the timely transfer of patients from the operating theatre to the Post-anaesthesia Care Unit and, subsequently, to the ward. This review aims to evaluate the available literature on MAS and FTC and compare their effectiveness. It was found that FTC is more appropriate for outpatient or day surgery procedures where rapid throughput and patient comfort are a priority. MAS, in itself, is very good for a low-income country like India. However, the addition of FTC can only enhance patient care if resources are made available. MAS can ensure consistency and efficiency in the discharge process, while using FTC can address broader recovery-related indicators and improve patient care. More research and modifications are further necessary.

## Introduction and background

Post-anaesthesia Care Unit (PACU), which was first established in 1923, is now recognised globally as the standard of care for quick recovery following surgery. Although it is a continuous process, patient recovery can be split into three different stages. Phase I of early recovery begins when anaesthesia stops being administered and continues until the patient regains their motor function and protective reflexes. After patients are moved from the PACU to a hospital ward or day surgery unit (DSU) until they are "home ready," intermediate recovery (phase II) starts. Phase III of late recovery continues until the patients are completely recovered. Regular breathing, an awake patient, hemodynamic stability, suitable oxygen saturation, and sufficient motor activity are indicators of an early surgical recovery [1-3]. After achieving this, the patient is transferred to a phase II recovery step-down unit, where they are monitored and made ready to return home [4,5].

The majority of Indian institutions use a single PACU that employs the time-based discharge protocol. When compared to conventional time-based discharge methods, criteria-based discharge scoring systems are more efficient with respect to both time and resources. This further aids in making the best use of the time and resources that are available [6-8]. There are two commonly used scoring systems to evaluate recovery from general anaesthesia (GA): the Modified Aldrete Score (MAS) and the Fast-Track Criteria (FTC). The most popular criterion for determining recovery is the MAS, which takes into account the patient's level of consciousness, activity, respiration, blood pressure, and oxygen saturation. Each of the five categories receives a score between 0 and 2, with a maximum score of 10 [9,10].

Recently, concerns have been expressed about the safety and effectiveness of the MAS due to its exclusion of an assessment for pain or nausea, which can exacerbate recovery from surgery and anaesthesia and cause many patients to experience these side effects. Prescription drugs with side effects like sedation leading to hypoventilation, nausea, and vomiting can also negatively impact post-operative recovery profiles [11-13]. A recovery assessment tool with built-in pain and nausea assessment was deemed necessary due to the rising number of laparoscopic surgeries where patients are released early, allowing medical professionals to feel confident in their discharge decisions securely. As a result, the FTC was created, which evaluates post-operative nausea vomiting (PONV), and pain in order to assess recovery from GA [14-16].

Nevertheless, there is currently little information on the comparison of the two scores to forecast recovery from GA following laparoscopic procedures, and this new criterion is not frequently employed in Indian hospitals. In addition to being more aware of the duration it takes to recover from GA, it's critical to comprehend how recovery scores from laparoscopic procedures help with prompt patient transfers from the operating room to the PACU and the ward [17,18].

## Review

Methodology

**Figure 1:**
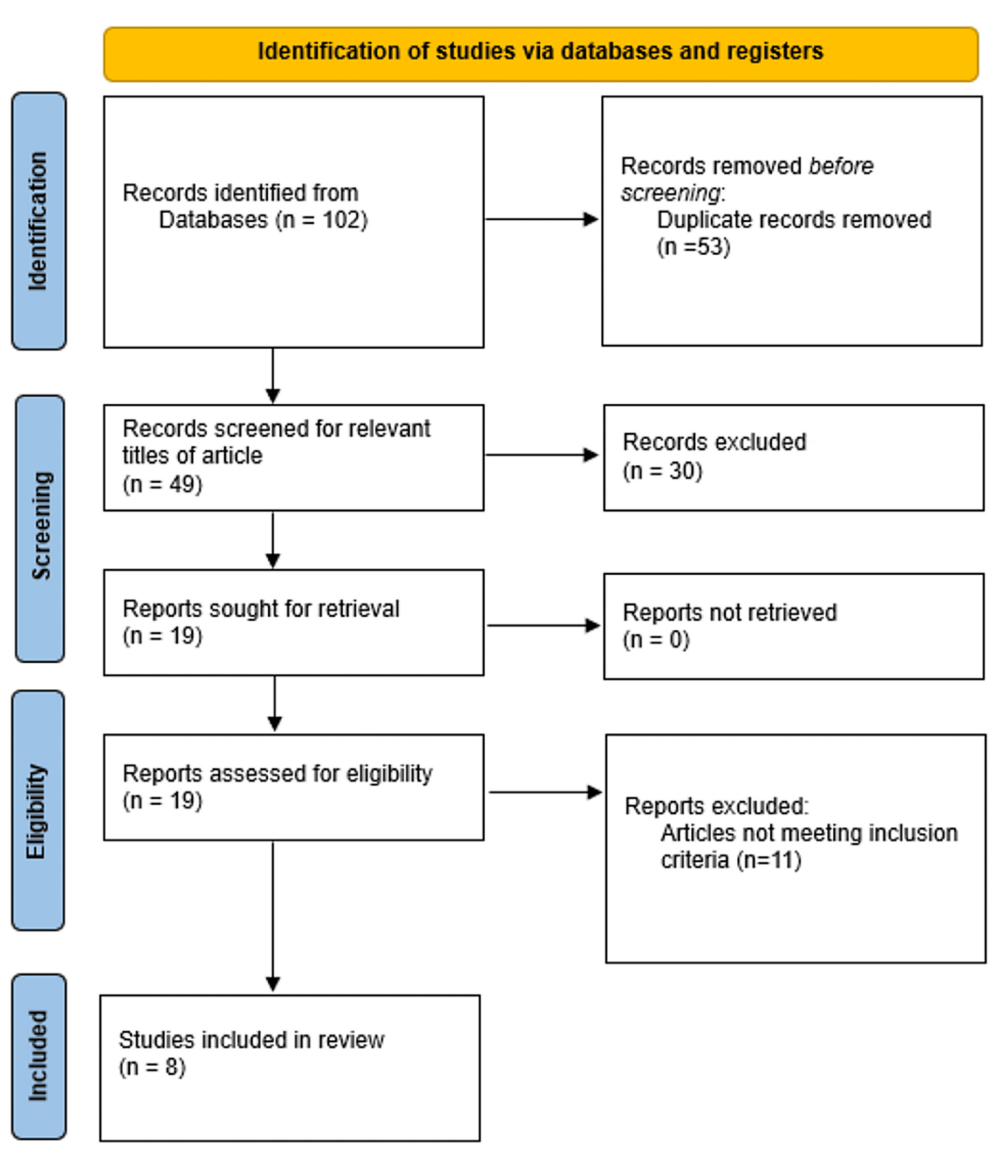
PRISMA flowchart PRISMA: Preferred reporting items for systematic reviews and meta-analyses

A search was conducted from September 1999 to October 2024 on electronic databases (PubMed, Google Scholar) using the search terms "Modified Aldrete Score" and "Fast-Track Criteria" in the abstract or title. During the search, study design criteria, publication type, and language limitations were applied. Inclusion criteria for the study included non-randomised trials, randomised controlled trials (RCTs), and studies that looked at post-anaesthesia recovery, MAS, and FTC published in English in peer-reviewed journals. Studies unrelated to the investigation or published in non-peer-reviewed journals were eliminated. RCTs, experimental studies, literature reviews, and other study designs were all utilised. Following a preliminary investigation, 102 articles were found in the search database; we then eliminated 53 articles that were duplicates. Thirty were excluded because they were irrelevant to the topic. After reviewing the full text of 19 articles, we excluded 11 because they did not meet the inclusion criteria. Eight articles were included in the final review. Figure 1 shows a summary of the selected publications based on the Preferred Reporting Items for Systematic Reviews and Meta-Analyses (PRISMA) guidelines.

Over the years, many discharge assessment tools have been developed to evaluate patient recovery and readiness for discharge from the PACU. However, more data is needed to compare these scoring criteria in low-income countries like India. Understanding how these scores can be effectively utilised in our settings is crucial for ensuring the timely transfer of patients from the operating theatre to the PACU and, subsequently, to the ward.

Banerjee et al. conducted a study in 2018 to evaluate and compare the MAS and FTC for recovery from GA in patients undergoing laparoscopic surgery. The drugs used were midazolam and fentanyl. According to MAS (score ≥9) and FTC (score ≥12), the recovery time from anaesthesia was determined to be 14.8 ± 3.8 and 13.0 ± 3.5 minutes, respectively. The recovery time difference was 1.75 minutes on average. While the MAS scores stayed unchanged, a decrease in the FTC scores was noted in 7% and 3% of the subjects at two and six hours after extubation, respectively, which is consistent with the study subjects' occurrence of severe post-operative pain and nausea/vomiting [19].

White and Song evaluated 216 female patients undergoing laparoscopic tubal ligation and cholecystectomy for recovery times, divided into three different groups (desflurane, sevoflurane, and propofol). The MAS and FTC showed a mean difference in recovery times of 1.2 minutes in the propofol group. The authors discovered advantages with FTC compared to the MAS criteria. The new fast-track scoring system accounts for Aldrete’s assessments of consciousness, physical activity, hemodynamic and respiratory stability, as well as pain and emetic symptoms. Significantly fewer outpatients would need intravenous (IV) medication in the step-down unit following laparoscopic surgery if the new FTC were followed [2].

Aggarwal et al., in 2024, conducted a study on 175 patients who were anaesthetised using propofol (IV) and maintained with sevoflurane and nitrous oxide. The author compared FTC, Traditional Time-Based, and MAS criteria and discovered that, when using the MAS, most patients had shorter stays in the PACU and left earlier than when using the FTC and Traditional Time-Based criteria. When compared to Time-Based criteria and FTC scoring, the MAS indicates an earlier rate of recovery and a shorter length of stay in the PACU [20]. Emmanni evaluated 80 patients for recovery post-anaesthesia using FTC or the MAS. When measured by MAS (score ≥9) and FTC (score ≥12), the mean time to recover from anaesthesia was found to be 20.5 ± 5.41 and 16.88 ± 6.95 minutes, respectively. Twelve per cent of the subjects showed a decline in their FTC scores between four and six hours after extubation. In contrast, their MAS scores did not change, which was consistent with the prevalence of severe post-operative pain, nausea, and vomiting in the trial patients [21,22].

Due to the limited available literature, some other studies were also evaluated. Burke and Kyker evaluated 73 adults referred for surgery under GA. Before leaving the operating room, patients were assessed using the MAS, FTC, and Speed Criteria by the anaesthesiologist, and then 5, 10, 15, and 30 minutes after arrival by the PACU nurse in the post-surgical suite. The authors discovered that when determining which patients will need phase I nursing interventions, the Speed Criteria are noticeably more accurate and sensitive. Truong et al. found that a modified clinical scoring system based on Aldrete’s score significantly reduced PACU length of stay compared to Time-Based criteria, suggesting productivity benefits in day surgery. Yamaguchi et al. found that patients evaluated with the MAS recovered faster and were discharged earlier compared to those assessed with the Modified Post-anaesthetic Discharge Scoring System (MPADSS). However, they experienced higher rates of drowsiness at discharge [23-25]. Table 1 shows the summary of articles reviewed for the analysis of MAS and FTC.

**Table 1:** Summary of articles reviewed for analysis of MAS and FTC PACU: Post-anaesthesia care unit, MAS: Modified Aldrete scoring, FTC: Fast-track criteria, PONV: Post-operative nausea vomiting, MPADSS: Modified post-anaesthetic discharge scoring system, LOS: Length of stay

Author name and year	Patient population	Study group	Methods	Author’s perspective
Banerjee et al. (2018) [19]	Patients undergoing laparoscopic surgery	100	All enrolled patients’ recovery was evaluated using both the FTC and the MAS. Scores were taken at 5-minute intervals up until 30 minutes after tracheal extubation.	In this context, the FTC and the MAS both seem appropriate for evaluating recovery in the early post-operative phase. Nonetheless, FTC ought to receive a higher rating because it adds records of PONV and post-operative discomfort.
White and Song (1999) [2]	Patients undergoing laparoscopic tubal ligation or cholecystectomy	216	Following the cessation of the maintenance anaesthetics, the early recovery status was assessed at 1 minute using the new FTC system in addition to the MAS system. Time intervals of 1 minute to 5 minutes after the patient’s arrival in the PACU were recorded, and then 5 minutes at a time until the patient attained FTC eligibility using both scoring systems. This process was repeated until the maintenance anaesthetics were stopped.	When determining whether an outpatient is suitable to bypass PACU treatment following ambulatory surgery under general anaesthesia, the new FTC scoring system appears to have advantages over the MAS system.
Emmanni (2019) [21]	Patients undergoing laparoscopic surgery	80	All enrolled patients’ recovery was evaluated using either the FTC or the MAS. Up until 30 minutes after tracheal extubation, scores were recorded every 5 minutes. The point in time at which a score of at least nine in the MAS and at least twelve in the FTC is achieved was to be noted. At 2, 6, 12, and 24 hours after tracheal extubation, the scores were taken.	When evaluating the recovery from general anaesthesia following laparoscopic surgery in the early post-operative phase, FTC and MAS appear to be about equally effective. FTC, on the other hand, offers an evaluation of PONV and is, therefore, more useful for recording sufficient recovery for patients being transferred from the PACU to the ward.
Burke and Kyker (2013) [26]	Patients who had general anaesthesia during surgery	73	Prior to shifting the patient from the operating room, the patients were assessed using the MAS, FTC, and speeds criteria. Five, ten, fifteen, and thirty minutes after entering the recovery area, the patients were evaluated again.	It is apparent that speeds have advantages over the FTC system and MAS in evaluating suitability for phase I recovery bypass following general anaesthesia because the requirements are self-explanatory and require a yes/no response without calculations for deviations from pre-operative blood pressure. Additionally, the Speed Criteria are significantly more sensitive and accurate in identifying patients who will require phase I nursing interventions.
Maqbool and Shahani (2012) [27]	Patients who had general anaesthesia during surgery	199	Patients were assessed for their recovery from general anaesthesia in the operating room using the FTC score (1, 2, 3). After being moved to the PACU, patients’ oxygen saturation levels were recorded immediately on air, and supplemental oxygen was administered. The patients’ further recovery was evaluated using the MAS system, which was used immediately, as well as at five, fifteen, thirty, and one-hour intervals, depending on the patient’s clinical physiological status and the score attained.	To standardise clinical recovery endpoints that span the wide range of co-morbid conditions of surgical patients throughout the performance of clinical research, simple recovery scoring systems are required. For the best possible patient care, the FTC criteria and the MAS system can provide trustworthy direction for assessing the physical state of a patient after surgery as they recover from surgical anaesthesia.
Aggarwal et al. (2024) [20]	Patients who had general anaesthesia during surgery	375	Every patient in the PACU was evaluated using MAS, FTC, and Traditional Time-Based criteria every five minutes until thirty minutes went by, and then every two, six, twelve, and twenty-four hours after tracheal extubation. The amount of time needed to meet the FTC requirements with a score of ≥12 and the MAS with ≥9 was noted.	The patient must first recover sufficiently before being moved from the operating room to the PACU and, finally, the ward. When compared to Time-Based criteria and FTC scoring, the MAS demonstrates early recovery and shortens the LOS in the PACU.
Truong et al. (2004) [23]	Patients who had general anaesthesia during surgery	400	In the PACU, a prospective cohort analysis evaluated the effectiveness of time-based discharge criteria (Group 1) against a modified clinical scoring system (Group 2) and the assessment of pain and temperature.	Aldrete’s scoring system can be easily, impartially, and clinically modified. As a result, patients would be free to follow the criteria and complete the recovery process at their own pace. Using our modified discharge criteria, the adjusted analysis demonstrated a significant reduction in PACU-LOS, which was not evident in the initial analysis. Its use in a day surgery setting might demonstrate a higher effect on productivity.
Yamaguchi et al. (2022) [24]	Patients who underwent gastrointestinal endoscopy under midazolam sedation	376	The MPADSS was used to evaluate 181 outpatients’ discharge status following sedated endoscopy (Group M). A group of 195 patients were evaluated using the MAS.	Group A exhibited a considerably larger number of patients who recovered within 60 minutes following endoscopy compared to Group M. Group A’s percentage of patients who needed more than 120 minutes to recuperate following an endoscopy was considerably lower than Group M’s. On the other hand, Group A’s discharge rate of drowsiness was much higher than Group M’s. Compared to patients evaluated using the MPADSS, patients evaluated using the MAS were permitted to be discharged earlier.

Discussion

Increased patient loads, staffing shortages, and space constraints have put more strain on post-operative intensive care unit (ICU) staff in developing nations like India [1]. As ambulatory anaesthesia becomes more common, it is crucial to establish criteria that will ensure patients are discharged more quickly and safely. The period immediately after the patient is moved to the PACU is the most critical for recovery because this is when the patient needs to be closely monitored in order to aid in the early detection of surgical complications. The MAS, which is most frequently used to assess discharge preparedness from the PACU, is being questioned for its effectiveness as a scoring tool because it ignores the most prevalent post-operative symptoms of pain, nausea, and vomiting. As a result, the FTC scoring system, which includes an evaluation of pain and emesis along with all the components of the MAS, has been used [28-30]. The analysis of a variety of discharge assessment tools addressing patient outcomes and discharge readiness in the PACU demonstrates the wide range and complexity of criteria used in various contexts and patient types. Numerous studies have assessed the efficacy of these tools, such as the MAS, FTC, and other scoring systems.

Banerjee et al. and Emmanni reported that the MAS and their FTC are effective for early post-operative evaluation and suggested that the FTC could have additional patient-related benefits by taking into consideration PONV and pain since they complete a comprehensive recovery evaluation [19,21]. These results indicate that a wider array of clinical parameters can be included to improve the effectiveness of the assessment for timely and safe PACU discharge. White et al. and Aggarwal et al. further emphasised the use of FTC, rather than Traditional Time-Based criteria and MAS, in specific outpatient surgical procedures. The addition of pain and emetic symptoms in the FTC scoring system enables a more detailed evaluation of recovery, which can reduce the necessity of early phase I nursing interventions and may shorten the discharge time.

Burke and Kyker assessed 73 patients using the MAS, FTC, and Speed Criteria. Evaluations were conducted pre-operatively and at various intervals post-PACU entry. The study found that the Speed Criteria had advantages over both the FTC and MAS, offering a simpler, more sensitive, and accurate method for evaluating suitability for phase I recovery, bypassing the need for complex calculations. The Speed Criteria's straightforward yes/no responses, without complex calculations, were particularly noted as beneficial. Maqbool and Shahani's study on 199 patients used the FTC in the operating room and the MAS in the PACU, with evaluations at multiple intervals post-operatively. The study underscored the need for simple recovery scoring systems that can standardise clinical recovery endpoints across diverse patient populations [26,27]. Both the FTC and MAS were found reliable for assessing the physical state of patients recovering from surgical anaesthesia. Aggarwal et al. evaluated 375 patients using the MAS, FTC, and Traditional Time-Based criteria at regular intervals post-operatively. The study indicated that the MAS demonstrated earlier recovery and reduced PACU length of stay compared to Time-Based criteria and FTC, suggesting its effectiveness in the initial recovery phase [20].

Truong et al.'s cohort analysis of 400 patients compared time-based discharge criteria with a modified clinical scoring system, including assessments of pain and temperature. The MAS system showed significant reductions in PACU length of stay, indicating its potential utility in day surgery settings to enhance productivity and patient throughput. Yamaguchi et al. compared the MAS and the MPADSS in 376 patients undergoing gastrointestinal endoscopy under midazolam sedation. The study found that patients evaluated with the MAS had faster recovery and earlier discharge, although with a higher rate of drowsiness compared to those evaluated with the MPADSS [23,24].

MAS is a simple and rapid, physiologically minimal, recovery-oriented score that may be well-suited for a typical PACU with limited resources. FTC adds the assessment of pain and PONV, which are associated with faster recovery times and earlier discharges. Studies in the United States also highlight its sensitivity to post-operative discomfort. FTC is more appropriate for outpatient or day surgery procedures where rapid throughput and patient comfort are a priority. MAS, in itself, is very good for a low-income country like India; however, the addition of FTC can only enhance patient care if resources are made available.

## Conclusions

Comparison of MAS with FTC to assess post-anaesthesia recovery shows advantages and limitations in this review. MAS is simple and effective in the evaluation of basic physiological recovery and is ideal for standard PACU rooms that could be richer in resources. On the contrary, FTC includes pain and PONV assessments as part of the evaluation, leading to earlier discharge and faster recovery times, particularly in outpatient and day surgery settings. MAS can ensure consistency and efficiency in the discharge process. If using FTC is feasible, it can address broader recovery-related indicators and enhance patient care. For post-anaesthesia care to be optimised and patient outcomes to be improved globally, more research and modification of these criteria in various clinical settings are necessary.
